# The maturation state of the auditory nerve and brainstem in rats exposed to lead acetate and supplemented with ferrous sulfate

**DOI:** 10.1016/j.bjorl.2016.12.004

**Published:** 2017-01-23

**Authors:** Fernanda Zucki, Thais C. Morata, Josilene L. Duarte, Maria Cecília F. Ferreira, Manoel H. Salgado, Kátia F. Alvarenga

**Affiliations:** a Universidade de São Paulo (USP), Faculdade de Odontologia de Bauru, Bauru, SP, Brazil; b National Institute for Occupational Safety and Health (NIOSH), Atlanta, USA; c Universidade de São Paulo (USP), Faculdade de Odontologia de Bauru, Departamento de Fonoaudiologia, Bauru, SP, Brazil; d Universidade Estadual Paulista “Júlio de Mesquita Filho” (UNESP), Departamento de Engenharia de Produção, Bauru, SP, Brazil

**Keywords:** Hearing, Auditory evoked potentials, Lead acetate, Blood lead levels, Ferrous sulfate, Audição, Potenciais evocados auditivos, Acetato de chumbo, Níveis de chumbo no sangue, Sulfato ferroso

## Abstract

**Introduction:**

The literature has reported the association between lead and auditory effects, based on clinical and experimental studies. However, there is no consensus regarding the effects of lead in the auditory system, or its correlation with the concentration of the metal in the blood.

**Objective:**

To investigate the maturation state of the auditory system, specifically the auditory nerve and brainstem, in rats exposed to lead acetate and supplemented with ferrous sulfate.

**Methods:**

30 weanling male rats (*Rattus norvegicus*, Wistar) were distributed into six groups of five animals each and exposed to one of two concentrations of lead acetate (100 or 400mg/L) and supplemented with ferrous sulfate (20 mg/kg). The maturation state of the auditory nerve and brainstem was analyzed using Brainstem Auditory Evoked Potential before and after lead exposure. The concentration of lead in blood and brainstem was analyzed using Inductively Coupled Plasma-Mass Spectrometry.

**Results:**

We verified that the concentration of Pb in blood and in brainstem presented a high correlation (*r* = 0.951; *p* < 0.0001). Both concentrations of lead acetate affected the maturation state of the auditory system, being the maturation slower in the regions corresponding to portion of the auditory nerve (wave I) and cochlear nuclei (wave II). The ferrous sulfate supplementation reduced significantly the concentration of lead in blood and brainstem for the group exposed to the lowest concentration of lead (100 mg/L), but not for the group exposed to the higher concentration (400 mg/L).

**Conclusion:**

This study indicate that the lead acetate can have deleterious effects on the maturation of the auditory nerve and brainstem (cochlear nucleus region), as detected by the Brainstem Auditory Evoked Potentials, and the ferrous sulphate can partially amend this effect.

## Resumo

**Introdução:**

A literatura relatou a associação entre o chumbo e os efeitos auditivos, com base em estudos clínicos e experimentais. No entanto, não há consenso em relação aos efeitos do chumbo no sistema auditivo, ou sua correlação com a concentrção do metal no sangue.

**Objetivo:**

Investigar o estado de maturação do sistema auditivo, especificamente do nervo auditivo e do tronco encefálico, em ratos expostos ao acetato de chumbo e suplementados com sulfato ferroso.

**Método:**

30 ratos machos desmamados (*Rattus norvegicus*, Wistar) foram distribuídos em seis grupos de cinco animais e expostos a urna de duas concentrações de acetato de chumbo (100 ou 400mg/L) e suplementados com sulfato ferroso (20mg/kg). O estado de maturação do nervo auditivo e do tronco encefálico foi analisado pelo Potencial Evocado Auditivo do Tronco Encefálico antes e após a exposição ao chumbo. A concentração de chumbo no sangue e tronco encefálico foi analisada utilizando-se Espectrometria de Massa com Plasma Indutivamente Acoplado.

**Resultados:**

Verificamos que as concentraçães de Pb no sangue e no tronco encefálico apresentaram alta correlação (*r* = 0.951, *p* < 0.0001). Ambas as concentraçõs de acetato de chumbo afetaram o estado de maturação do sistema auditivo, sendo a maturação mais lenta nas regiões correspondentes á porção do nervo auditivo (onda I) e núcleos cocleares (onda II). A suplementação com sulfato ferroso reduziu significativamente a concentração de chumbo no sangue e tronco cerebral no grupo exposto á menor concentração de chumbo (100 mg/L), mas não para o grupo exposto á maior concentração (400 mg/L).

**Conclusão:**

Esse estudo indica que o acetato de chumbo pode ter efeitos deletérios na maturação do nervo auditivo e do tronco encefálico (região do núcleo coclear), como detectado pelos potenciais evocados auditivos do tronco encefálico, e que o sulfato ferroso pode diminuir parcialmente esse efeito.

## Introduction

A significant association between higher lead (Pb) exposure and negative developmental outcomes including intelligence, cognitive, emotional and behavioral problems has been reported. Recently, low-level Pb exposure has been shown to have small, but significant negative impacts that persist across childhood and early adolescence, and into early adulthood.

The literature has also reported the association between Pb and auditory effects, based on clinical and experimental studies. However, there is no consensus regarding the effects of Pb in the auditory system, or its correlation with the concentration of the metal in the blood.^1–6^

Given Pb’s general toxicity in humans, some studies aimed to examine the protective effects of chelating agents, antioxidants, among others.^7–9^ A study^8^ presented a review about the beneficial effects of different antioxidants in preventing Pb body burden and oxidative stress. It finds that the administration of natural or synthetic antioxidants has been shown to be of benefit in the prevention and attenuation of metal induced biochemical alterations, but the human studies are still limited in this regard. In studies that have investigated the possible protective effects of iron (Fe) on Pb exposure, it was found that Fe prevented cytotoxicity and apoptosis induced by Pb,^10^ possibly protecting the integrity of the blood brain barrier.^11^ In contrast, previous studies that examined the effect of Pb on the brain antioxidant system and the central auditory system, did not confirm a protective effect of Fe in the auditory cortex.^12,13^

Brainstem Auditory Evoked Potential (BAEP) is an electrophysiological measure that has been used in research involving Pb exposure and auditory system. It measures the electrical activity that occurs from the distal portion of the auditory nerve to the inferior colliculus in the brainstem region, mapping the synapses of the auditory pathways related to the cochlear nerve, cochlear nuclei, superior olivary complex (bridge) and inferior colliculus (midbrain). The BAEP represents the sequential activation of a series of nuclei, expressed in the appearance of a wave; the change in its amplitude may indicate the particular activation of brainstem nuclei and the development of peripheral-central auditory pathway, showing the correlation between the development of synaptic function and postnatal maturation potential of this process.^14^ The latency indicates the time elapsed between the presentation of the acoustic stimulus and the occurrence of the peaks. As the auditory system matures, there are gradual changes in the morphology of the BAEP and in latency and amplitude characteristics of the various BAEP peaks. These changes have been attributed to the progressive myelination of fibers, better neural synchronization, and increased efficiency of synaptic transmission within the auditory pathway.^14–16^ In humans, the retro-cochlear and brainstem pathways mature from birth until the second year of life.^17^

Electrophysiological studies in humans or animals exposed to Pb were conducted in sample with a mature peripheral and central auditory system (up to brainstem), not allowing the evaluation of the relationship between Pb intoxication and the maturation state of the auditory system.

The purpose of the present study was to analyze the maturation state of the auditory nerve and brainstem in rats exposed to lead acetate, as well as if the administration of ferrous sulfate prevented the adverse effects of lead acetate.

## Methods

This study was approved by Ethics Committee for Animal Experiments of the Faculdade de Odontologia de Bauru/Universidade de São Paulo - FOB/USP, Brazil (process number 019/2009). A pilot study was conducted to establish the relationship between the administered lead acetate in water and the obtained Pb concentration in the blood of animals. Two concentrations of lead acetate in water were established, 100 and 400 mg/L, representing low and high exposure to Pb, respectively. Concentrations of Pb in the blood of animals of ~10 μg/dL and 30 μg/dL were obtained.

### Animals and exposure

The study was conducted with 30 weanling male rats (Rattus norvegicus, Wistar), aged 26 days and weighed on average 96–105 g each. They were bred by the proponent university. The animals were divided into six groups of five animals each:

- Control: received only deionized drinking water;
- 100 mg/L Pb: received 100 mg/L of Pb (CH_3_COO)_2_ in drinking water;
- 100 mg/L Pb + FeSO_4_: received 100 mg/L of Pb (CH_3_COO)_2_ in drinking water and a solution of FeSO_4_ at a dose of 20 mg/kg body weight;
- 400 mg/L Pb: received 400 mg/L of Pb (CH_3_COO)_2_ in drinking water;
- 400 mg/L Pb + FeSO_4_: received 400 mg/L of Pb (CH_3_COO)_2_ in drinking water and a solution of FeSO_4_ at a dose of 20 mg/kg body weight;
- FeSO_4_: received drinking deionized water, and a solution of FeSO_4_ at a dose of 20 mg/kg body weight.

The animals were kept for six weeks in metabolic cages in a temperature and humidity controlled room with 12 h light and 12 h of dark cycles, with food and water *ad libitum*. The diet selected was the Purina^®^ (Labina-Purina, São Paulo, SP, Brazil) commercial feed, which has low Pb and Fe contents. The drinking water was prepared by dissolving lead acetate in distilled and deionized water at concentrations of 100 mg/L and 400 mg/L, and the concentration of Pb in water was analyzed by Atomic Absorption Spectrometry in Graphite Furnace. Water consumption was evaluated every 2 days.

The ferrous sulfate (FeSO_4_) solution and the deionized water were administered once every two days to the animals orally by gavage, using a rigid stainless steel tube with the rounded tip. Gavage is considered the most accurate method of oral administration for the animal, in addition, to allowing the administration of the predetermined exact dosage. Even so, we tried to control possible losses of solution calculating the remaining amount of solution in the tube, making sure that the initially established contents were administered as well as the gavage being performed by an experienced professional.

### Brainstem Auditory Evoked Potentials - BAEP

The BAEP was conducted before and after Pb exposure, when the rats were 26 days old and repeated when they turned 63 days old, in order to evaluate the maturation state of their auditory system.

### Preparation of the animal

Each rat was lightly sedated with Ketamine and Xylazine (substances that do not interfere with test responses) for the placement of surface electrodes used to record the evoked potentials. We performed the trichotomy of the skull and skin cleansing, applying the electrolytic paste in the surface Embramac electrodes. These were connected to the preamplifier EPA 25, and positioned as follows: active electrode in Fz, reference electrodes positioned in M1 and M2 (left and right mastoid), and ground electrode on the back foot. The electrode impedances were kept lower than 5 kΩ and impedance between them lower than 2 kΩ.

### Brainstem Auditory Evoked Potentials recording

BAEP was obtained using an equipment Smart-EP by Intelligent Hearing Sistems (IHS) (Miami, FL, USA) to generate acoustic stimulation and for signal acquisition. The test ear was randomly selected. Click stimuli (rarefaction) was presented unilaterally through an earphone (pulse duration, 0.1 ms; presentation rate, 21.1 click/s) at an intensity of 80 dB nHL, and averaged across 1000 presentation. The responses were filtered with a band-pass filter of 100–3000 Hz.

We obtained two records for each ear and the presence of response was considered by observing the reproducibility of the waves. We analyzed the absolute latencies of waves I-IV and the values of interpeak intervals. This study did not consider the V wave as the occurrence of this wave can be considered atypical, even in normal rats.^18^

### Collection of samples

At the end of the experimental period the animals were anesthetized with ketamine hydrochloride and xylazine. The peritoneal cavity and chest were exposed, and the heart was punctured for blood collection in tubes Trace Royal Blue BD Hemogard (BD Vacutainer^®^, USA), that present low levels of trace elements, and stored at −20 °C for later analysis of Pb. The brainstem of the animal was dissected, weighed and immediately frozen in liquid nitrogen and stored at −80 °C for later analysis of Pb concentration in the tissue.

### Analysis of lead in whole blood and brainstem

The analysis of Pb in blood and brainstem were performed in a mass spectrometer with inductively coupled plasma was used; it was equipped with a reaction cell (DRC-ICP-MS ELAN DRCII, Perkin Elmer, Sciex, Norwalk, CT, EUA), as described in Batista et al.^19^ For tissue samples and Certified Reference Materials (CRMs), approximately 75 mg were weighed into 15 mL polypropylene Falcon^®^ tube (Becton Dickinson). Then, 1 mL solution of tetramethylammonium hydroxide (TMAH) 50% (v/v) was added to the samples and incubated at room temperature for 12 h on a rotating homogenizer. After solubilization, the volume was adjusted to 10 mL with a dilute solution containing nitric acid (HNO_3_) 0.5% (v/v), 10 μg/L Rh and Triton X-100 0.01% (v/v). Analytical calibration standards were prepared in concentration range between zero and 20 μg/L, in a diluent containing TMAH 5% (v/v), HNO_3_ 0.5% (v/v), 10 μg/L Rh and Triton X-100 0.01% (v/v). The detection limit for Pb was 0.0066 μg/L.

For the blood analysis 200 μL of each sample were placed in a polypropylene Falcon^®^ tube of 15 mL (Becton Dickinson), striking up to a final volume of 10 mL (50 fold dilution) with a solution containing HNO_3_ 0.5% (v/v), 10 μg/L Rh and Triton X-100 0.01% (v/v). Analytical calibration standards were prepared in the concentration range between zero and 20 μg/L in the same diluent. The curve was made by setting the matrix, which contain those calibration standards, sheep blood diluted 50 times (based blood) and diluent.

Samples were prepared and directly injected into the equipment. Quality control for the analysis of Pb followed the guidelines provided in reference materials by the National Institute of Standards and American Technology (NIST 955c), by the Department of Health of New York State (NYSDOH-proficiency testing program for trace elements in whole blood) and by the National Public Health Institute of Quebec, Canada (INSP-analysis scheme for external quality (EQAS) for trace elements in blood).

### Statistical analysis

For data analysis we used the GraphPad Instat software (version 3.0 for Windows, GraphPad Inc. La Jolla, CA, USA software). Initially we checked the normality (Kolmogorov-Smirnov) and homogeneity of the data (Bartlett’s test), for selecting the appropriate statistical test.

The Pb concentration in the blood was analyzed using ANOVA followed by the Tukey test, after logarithmic transformation. Then, the concentration of Pb in the brainstem was analyzed using the Kruskal-Wallis test followed by Dunn’s test for individual comparisons.

For the statistical analysis of BAEP we used SPSS software version 13.0. The paired *t* test was used for comparison of results between right and left ear. The ANOVA with two-factor repeated measures model was adopted to evaluate three variables:

Group Effect: pre and post-exposure to Pb results were compared between groups;
Time Effect: comparison of the results pre and post-exposure to Pb regardless of the group;
Group × Time Effect: comparison of the results pre and post-exposure to Pb by group and time.

## Results

### Blood and brainstem lead level

The administration of FeSO_4_ reduced the Pb concentration in blood and the brainstem (Table 1). For blood, the lowest Pb concentrations were observed in the control and the FeSO_4_ groups, while highest value was found to 400 mg/L Pb group. Concomitant administration of FeSO_4_ for the groups 100 mg/L Pb + FeSO_4_ and 400 mg/L Pb + FeSO_4_ reduced the concentration of Pb in the blood by about 65% and 50% respectively, however difference was observed only for 100 mg/L Pb and 100 mg/L Pb + FeSO_4_ (Table 1).

In the brainstem, a difference was observed between the control group and 400 mg/L Pb and between FeSO_4_ group with the groups 400 mg/L Pb and 400 mg/L Pb + FeSO_4_. While a reduction in the concentration of Pb in the brainstem of animals receiving concomitant administration of ferrous sulfate was observed, there was no difference when compared to their peers without supplementation, as shown in Table 1.

### Brainstem Auditory Evoked Potentials

The normality test of Kolmogorov-Smirnov showed normal distribution of the BAEP results obtained in this study (*p* ≤ 0.05). In the analysis of waves I-IV of BAEP pre and post-exposure to Pb, the ear factor was not considered individually, since when the right and left ears were compared (paired *t* test), and no difference was observed (*p* > 0.05).

**Figure 1:**
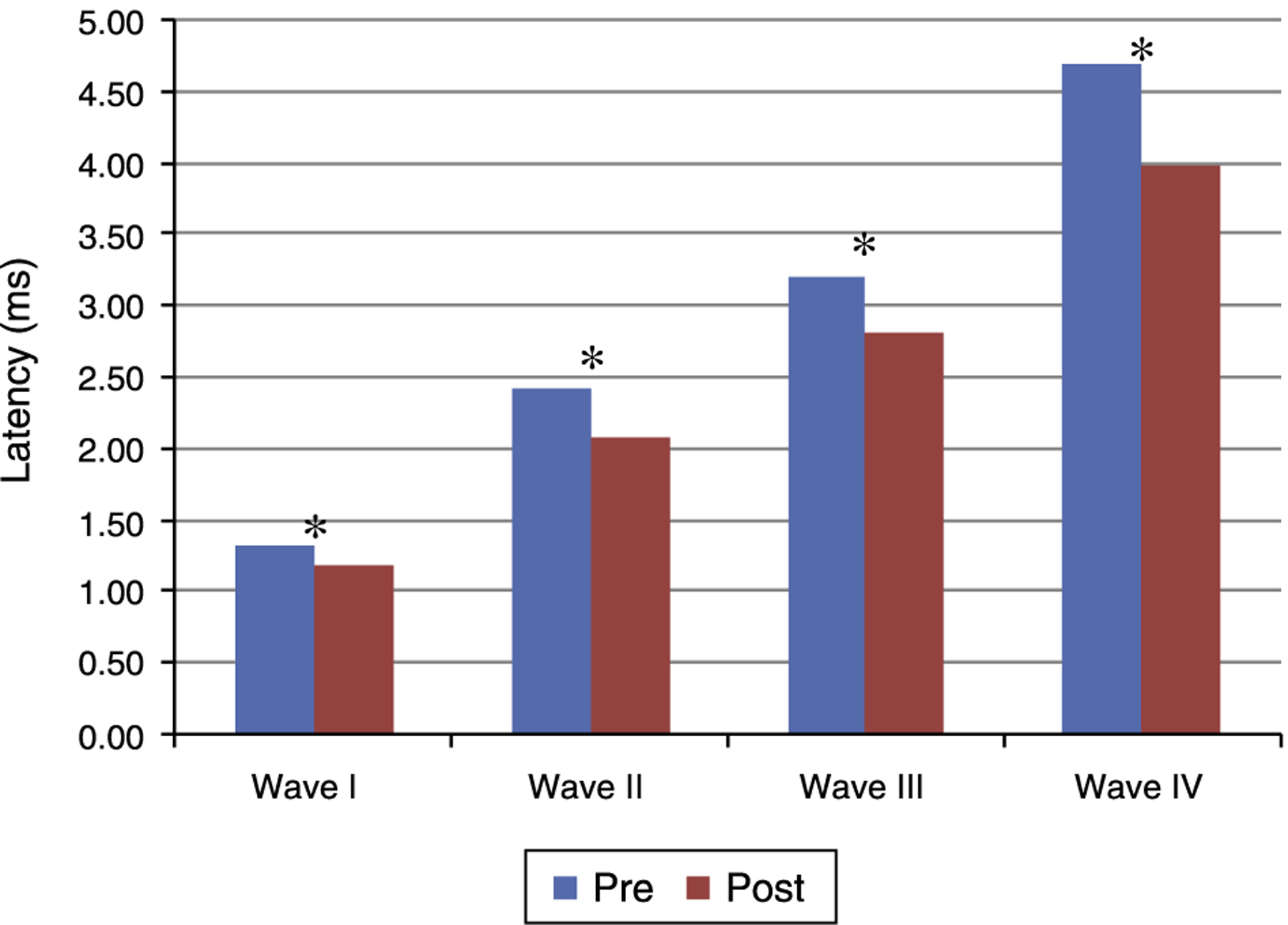
Mean and *p*-value of absolute latencies (in ms) of waves I-IV of BAEP for the Time Effect. Pre, Pre-exposure to Pb; Post, Post-exposure to Pb; ms, milliseconds; *p* obtained from the comparison at each moment (pre and post-exposure to Pb); * *p* ≤ 0.01; Pre-exposure to Pb, rats with 26 days old; Post-exposure to Pb, rats with 63 days old. The maturation of the auditory system (auditory nerve and brainstem) that occurs in humans from birth to the second year of life, promotes a gradual decrease in absolute latencies of BAEP waves.

**Figure 2:**
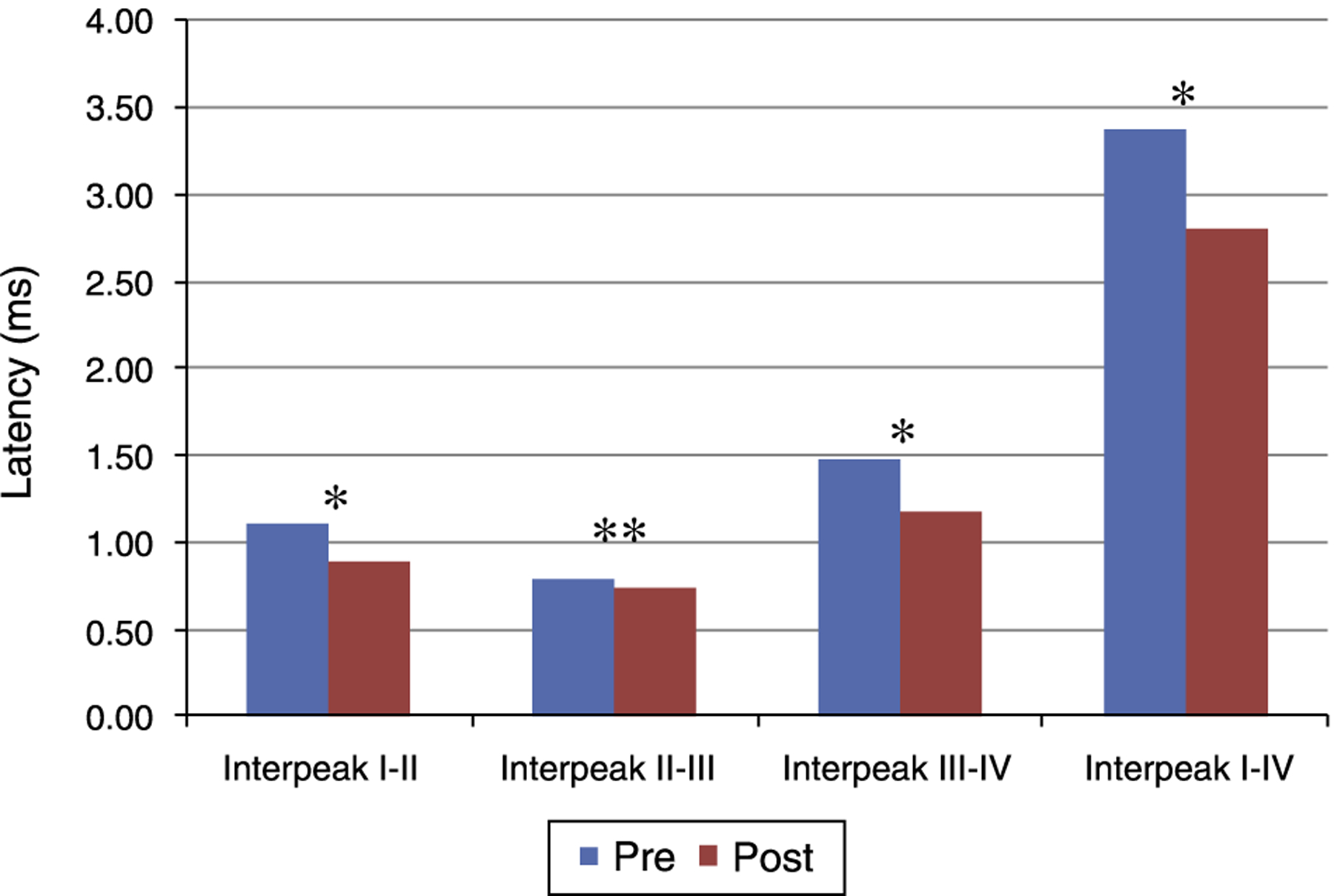
Mean and *p*-value of the interpeak intervals I-II, II-III, III-IV and I-IV for BAEP for the Time Effect. Pre, Pre-exposure to Pb; Post, Post-exposure to Pb; ms, milliseconds; *p*-obtained from the comparison at each moment (pre and post-exposure to Pb); * *p* ≤ 0.01; ** *p* ≤ 0.05; Pre-exposure to lead, rats with 26 days old; Post-exposure to Pb, rats with 63 days old. The maturation of the auditory system (auditory nerve and brainstem) that occurs in humans from birth to the second year of life, promotes a gradual decrease in absolute latencies of BAEP interpeak intervals.

Figs. 1 and 2 present the mean and the *p* value of absolute latencies of waves I-IV and the interpeak intervals I-II, II-III, III-IV and I-IV, respectively, for the Time Effect. The analysis of the Time Effect demonstrated a difference for all absolute latencies waves (Fig. 1) and interpeak intervals (Fig. 2) between the period pre and post-exposure to Pb was observed.

Tables 2 and 3 show the mean and *p*-value of absolute latencies of waves I-IV and the interpeak intervals I-II, II-III, III-IV and I-IV, respectively, for the Group Effect.

## Discussion

Different researchers have hypothesized mechanisms by which Pb may alter biochemical, pharmacological and molecular processes in the Central Nervous System (CNS),^20^ including (1) Pb interactions with cellular proteins^21^; (2) inhibition of voltage-sensitive calcium channels^22^; (3) perturbation of synaptic transmission of a number of neurotransmitters^22,23^; (4) alteration of the development and growth of neurons and astroglia in culture systems,^24^ and (5) direct interaction with essential metal ions in the brain.^25^

The Fe level in specific tissues in the CNS is regulated to meet the specialized needs of metabolism and prevent cytotoxicity.^26,27^ Fe absorption is controlled by a mechanism known as Fe homeostasis,^27^ and despite the importance of this homeostasis, the mechanisms involving the absorption of Fe in the brain are not well understood.^28^ The loss of homeostasis can occur from acute conditions such as neuronal dysfunction caused by stroke (when the blood brain barrier is compromised), or progressive neurological disorders such as Alzheimer’s, Parkinson’s, Huntington’s disease, hereditary disorders such as hemochromatosis and the accumulation of metals as Pb and cadmium in the choroid plexus.^20,29–32^ Selective choroid plexus toxicants usually do not directly alter choroid plexus’ permeability, nor do they produce massive pathophysiological alterations seen in clinics. However, the metals like Pb, on course to the brain, may selectively act on certain critical regulatory functions of the choroid plexus, giving rise to profound neurotoxic consequences.^20^ The Fe is considered a sequestered choroid plexus toxicants, which are also called barrier stored toxicants, and may deposit in or be sequestered by the choroid plexus. Nevertheless, the sequestration of these metals in the tissue has not been associated with any known pathological or pathophysiological consequences to the blood-cerebrospinal fluid barrier.^20^ Thus, we can suppose that the imbalance in Fe homeostasis in the brain caused by Pb administration can be reversed by Fe supplementation, reducing Pb absorption by brainstem.

In studies that have investigated the possible protective effects of Fe on Pb exposure, it was found that Fe prevented cytotoxicity and apoptosis induced by Pb,^10^ possibly protecting the integrity of the blood brain barrier.^11^ In contrast, other studies that examined the effect of Pb on the brain antioxidant system and the central auditory system, did not confirm a protective effect of Fe in the auditory cortex.^12,13^ Our observations demonstrated that ferrous sulfate had a protective effect toward Pb neurotoxicity. We verified a reduced blood Pb levels when ferrous sulfate was administered to animals exposed to Pb. The difference was statistically significant for the group that received the lowest dose of lead acetate. Previous studies used concentrations of ferrous sulfate that ranged from 7 to 40 mg/kg.^10,11^ One study reported that 7 mg/kg Fe decreased Pb burden in the brain and was protective while 14 mg/kg increased Pb in the brain but nevertheless seems to protect the blood-brain barrier. In a follow-up publication, they used 20 mg/kg as a low dose and 40 mg/kg Fe as a high dose and they reported exactly the same amount of Pb in the blood as in their previous study. These studies presented methodological inconsistencies that made the relationship between Fe supplementation and Pb associated effects remain unclear. For this reason, in the present study we used 20 mg/kg of ferrous sulfate that corresponds to an average value presented in the literature (ranged from 7 to 40 mg/kg)^10,11^ and our results demonstrated that 20 mg/kg was able to significantly reduce the Pb concentration in the blood.

The analysis of the concentration of Pb in the brainstem and in blood confirmed a high correlation between the concentration of Pb in them (*r* = 0.951, *p* < 0.0001). However, despite having verified a reduction in Pb concentrations in the brainstem tissue for the 100 mg/L Pb + FeSO_4_ and 400 mg/L Pb + FeSO_4_ groups, It was not significant when compared to the groups that do not receive ferrous sulfate. In a study^11^ that used only one concentration of lead acetate (342 mg/mL) associated with supplementation with two doses of ferrous sulfate (7 and 14 mg/kg), the analysis of Pb level In three different brain tissues demonstrated that the groups supplemented with low concentrations of ferrous sulfate had a significant reduction in the concentration of Pb in these regions compared with the group that received only lead acetate, while the high concentration of ferrous sulfate supplementation had no significant effect on levels of Pb in the brain.

Regarding the results obtained in BAEP, the analysis of the Time Effect (pre and post-exposure to Pb) demonstrated a significant reduction in the absolute latencies of waves I-IV (Fig. 1)and the values of interpeak intervals I-II, II-III, III-IV and I-IV (Fig. 2) in all groups. This result was expected since the study was developed in order to include the maturation state of the auditory nerve and brainstem, with the research of BAEP pre-exposure being performed in rats with 26 days old and research post exposure with 63 days of life. Thus, the results demonstrated that exposure to lead acetate did not preclude the maturation state of the auditory system structures, because there was a reduction in the absolute latency of waves and interpeak intervals of values with increasing age. This reflects the gradual myelination of fibers, neural synchronization and increased efficiency of transmission at the synapse within the nuclei in the auditory pathway of the brainstem.^14–16^

However, in the analysis of Group Effect, we observed that the control and 100 mg/L Pb + FeSO_4_ groups present differences in the absolute latency of wave III when compared to the other groups (*p* = 0.030) at the pre-exposure testing (Table 2). Similarly, the FeSO_4_ and 100 mg/L Pb groups had different values of I-II interpeak interval when compared to the other groups (*p* = 0.011) (Table 3). The results of the post-exposure testing revealed that the difference was not maintained; namely, the absolute latency of wave III and the values of I-II interpeak interval were not different among the groups (*p* = 0.189 and *p* = 0.131, respectively). The data suggests that the maturation state was slower in the auditory nerve and the cochlear nucleus (lower portion of the brainstem) in groups exposed to low concentrations of Pb.

Additionally, the analysis of the Group × Time Effect showed that the reduction expected in the value of interpeak interval I-II, was lower in the 100 mg/L Pb and 400 mg/L Pb (*p* = 0.049) when compared to the other groups. This reinforces the effect of Pb in the maturation of the peripheral auditory system structures, as wave I corresponds to the activity of auditory nerve and wave II corresponds to the region of the cochlear nucleus. The results obtained from the analysis of the Group Effect and Group × Time Effect, indicated that for both of the concentration of lead acetate used, the maturation state was slower in the regions corresponding of the auditory nerve and cochlear nuclei, in agreement with the results reported by other studies.^33,34^

The other studies reported in the literature that performed BAEP in humans and animals with a history of Pb poisoning, did not include the analysis of the maturation state of the peripheral and central auditory system (up to the brainstem). However, it is noteworthy that, the toxic effect of Pb in these structures of the system was confirmed in most studies.^1,35–45^ But there are descriptions of normal BAEP results,^2,46–49^ as well as lack of correlation between the results of BAEP and the concentration of Pb in blood.^6,49^

In the case of FeSO_4_ supplementation, the results indicated the strengthening of the initial hypothesis that this modifies the effects of the exposure to lead acetate. The slower maturation in the groups poisoned by lead acetate, independent of concentration, as discussed above, was not observed when FeSO_4_ was co-administered, since no difference in absolute latencies and interpeak intervals were found for groups administered with 100 mg/L Pb + FeSO_4_ and 400 mg/L Pb + FeSO_4_ in comparison to the control group. This is further strengthened by the results of the analysis of the Group Effect on the value of the interpeak interval I-IV, which showed a difference between groups 100 mg/L Pb and 100 mg/L Pb + FeSO_4_ in the period post-exposure (*p* = 0.033) (Table 3). When considering that the values interpeak interval in the pre-exposure period in these groups showed no difference (*p* = 0.143) (Table 3), it is possible to observe a slower reduction of the interpeak interval, and consequently the maturation state, in the unsupplemented group. Therefore, the results suggest a protective effect of ferrous sulfate on the toxicity of lead acetate in a specific region of the auditory system.

A study^45^ analyzed the impact of Pb on the auditory system of rats and guinea pigs and their molecular mechanisms, in addition to the protective action of Fe. Their results showed that Pb increased BAEP thresholds in animals, and FeSO_4_ when administered orally (gavage), reduced significantly the BAEP threshold increase by Pb-induced, thereby protecting the auditory system against toxicity induced by Pb. In studies that have investigated the possible protective effects of Fe on Pb exposure, it was found that Fe prevented cytotoxicity and apoptosis induced by Pb,^10^ possibly protecting the integrity of the blood brain barrier.^11^ In contrast, previous studies that examined the effect of Pb on the brain antioxidant system and the central auditory system, did not confirm a protective effect of Fe in the auditory cortex.^12,13^

We considered that our results must be analyzed with caution before conclud that Fe “protect” the BAEP from Pb effects. BAEP latencies are influenced, among many factors, by axon caliber, which is responsive to Fe levels. As baseline Fe levels and post-exposure Fe levels could not been obtained, it is possible that the “normalization” of the BAEP latency in the presence of Fe represents a combination of slower maturation caused by Pb and premature maturation caused by axon caliber size changes due to Fe. Both mechanisms together might lead to a seemingly “normal” BAEP latency. If that were the case, it would perhaps not be correct to call the response seen in the presence of Fe as “protective” since we do not know what role each mechanism led to the measured BAEP latencies in the first place nor do we know whether all features were restored to normal function.

## Conclusion

This study indicate that the lead acetate can have deleterious effect on the maturation state of the auditory nerve and brainstem (cochlear nucleus region), as detected by Brainstem Auditory Evoked Potentials and that the ferrous sulphate can partially amend this effect.

## Figures and Tables

**Table 1:** Mean (±SD) of blood lead level and brainstem of animals from control and experimental groups (*n* = 5/group).

Groups	[Lead] blood (μg/dL)	[Lead] brainstem (ng/g)
Control	0.19 ± 0.10	11.18 ± 4.42
100 mg/L Pb	10.56 ± 5.29^a,b^	27.21 ± 7.03
100 mg/L Pb + FeSO_4_	3.89 ± 2.04^a^	19.53 ± 9.41
400 mg/L Pb	48.39 ± 8.66^a^	259.88 ± 76.^a^
400 mg/L Pb + FeSO_4_	25.85 ± 3.61^a^	208.87 ± 91.10
FeSO_4_	0.13 ± 0.02	8.54 ± 1.34^c^

a Mean with significant difference when compared control with experimental groups.

b Mean with significant difference between the groups 100 mg/L Pb and 100 mg/L Pb + FeSO_4_.

c Mean with significant difference between the groups FeSO_4_, 400 mg/L Pb and 400 mg/L Pb + FeSO_4_.

**Table 2:** Mean and *p*-value of absolute latencies (in ms) of waves I-IV of BAEP for the Group Effect.

Group Effect (*n* = 30)	Waves (ms)							
	I Exposure		II Exposure		III Exposure		IV Exposure	
	Pre	Post	Pre	Post	Pre	Post	Pre	Post
Control	1.388	1.236	2.566	2.109	3.418	2.882	4.895	4.128
100 mg/L Pb	1.292	1.167	2.278	2.079	3.101	2.888	4.594	4.091
100 mg/L Pb + FeSO_4_	1.231	1.230	2.328	2.038	3.067	2.753	4.490	3.850
400 mg/L Pb	1.324	1.197	2.345	2.106	3.181	2.827	4.582	3.987
400 mg/L Pb + FeSO_4_	1.378	1.110	2.539	2.007	3.340	2.692	4.753	3.854
FeSO_4_	1.270	1.195	2.451	2.118	3.152	2.844	4.822	4.017
*p*	0.237	0.397	0.066	0.468	0.030^a^	0.189	0.175	0.125

Pre, Pre-exposure to Pb; Post, Post-exposure to Pb; ms, milliseconds.

a *p* obtained from the comparison of the groups at each time (pre and post-exposure to Pb).

**Table 3:** Mean and *p* value of interpeak intervals (in ms) I-II, II-III, III-IV and I-IV of BAEP for the Group Effect.

Group Effect (*n* = 30)	Interpeak intervals (ms)							
	I-II Exposure		II-III Exposure		III-IV Exposure		I-IV Exposure	
	Pre	Post	Pre	Post	Pre	Post	Pre	Post
Control	1.178	0.873	0.852	0.773	1.477	1.246	3.507	2.892
100 mg/L Pb	0.986	0.912	0.823	0.809	1.493	1.203	3.302	2.924
100 mg/L Pb + FeSO_4_	1.097	0.808	0.739	0.715	1.423	1.097	3.259	2.620
400 mg/L Pb	1.021	0.909	0.836	0.721	1.401	1.160	3.258	2.790
400 mg/L Pb + FeSO_4_	1.161	0.897	0.801	0.685	1.413	1.162	3.375	2.744
FeSO_4_	1.181	0.923	0.701	0.726	1.670	1.173	3.552	2.822
*p*	0.011^a^	0.131	0.384	0.200	0.192	0.270	0.143	0.033^a^

Pre, Pre-exposure to Pb; Post, Post-exposure to Pb; ms, milliseconds.

a *p* obtained from the comparison of the groups at each time (pre and post-exposure to Pb).
